# A Rare Case of Proximal Junctional Failure with Delayed Infection after Adult Spinal Deformity Surgery: A Report of Two Cases

**DOI:** 10.1155/2020/8883828

**Published:** 2020-07-10

**Authors:** Takuro Iwami, Mitsuru Yagi, Eijiro Okada, Satoshi Suzuki, Satoshi Nori, Osahiko Tsuji, Narihito Nagoshi, Kota Watanabe, Nobuyuki Fujita, Masaya Nakamura, Morio Matsumoto

**Affiliations:** ^1^Department of Orthopedic Surgery, Keio University School of Medicine, Japan; ^2^Department of Orthopedic Surgery, Fujita Health University, Japan

## Abstract

Proximal junctional failure (PJF) is one of the most devastating complications that develop after adult spinal deformity (ASD) surgery. Here, we report 2 rare cases of PJF accompanied by delayed infection after ASD surgery with a review of the relevant literatures. Late-onset infection is an infrequent complication despite acute postoperative infection is common after posterior spinal instrumentation and fusion. Among them, delayed onset pyogenic spondylitis of the adjacent vertebra to the instrumented vertebrae is an extremely rare phenomenon. We do not have a clear explanation for this pathology. Since the delayed infections developed not in the fused segments but in the adjacent vertebra, the cause of the first case can be speculated as stimulation of low-virulent organisms to fester and hematogenous seeding and that of the second case as metal fretting and a sterile inflammatory response causing hematogenous microbial seeding, respectively. Additional studies on this phenomenon are warranted to elucidate the pathogenesis of this complication.

## 1. Background

Proximal junctional failure (PJF) is one of the most devastating complications that occur after adult spinal deformity (ASD) surgery [1–3]. In patients with PJF, paralysis or severe pain can occur due to a fracture of the adjacent vertebra, loosening of the implant, or failure of the posterior ligamentous complex. Here, we report 2 rare cases of PJF accompanied by delayed infection after ASD surgery with a review of the relevant literatures.

## 2. Case

### 2.1. Case 1

**Figure 1:**
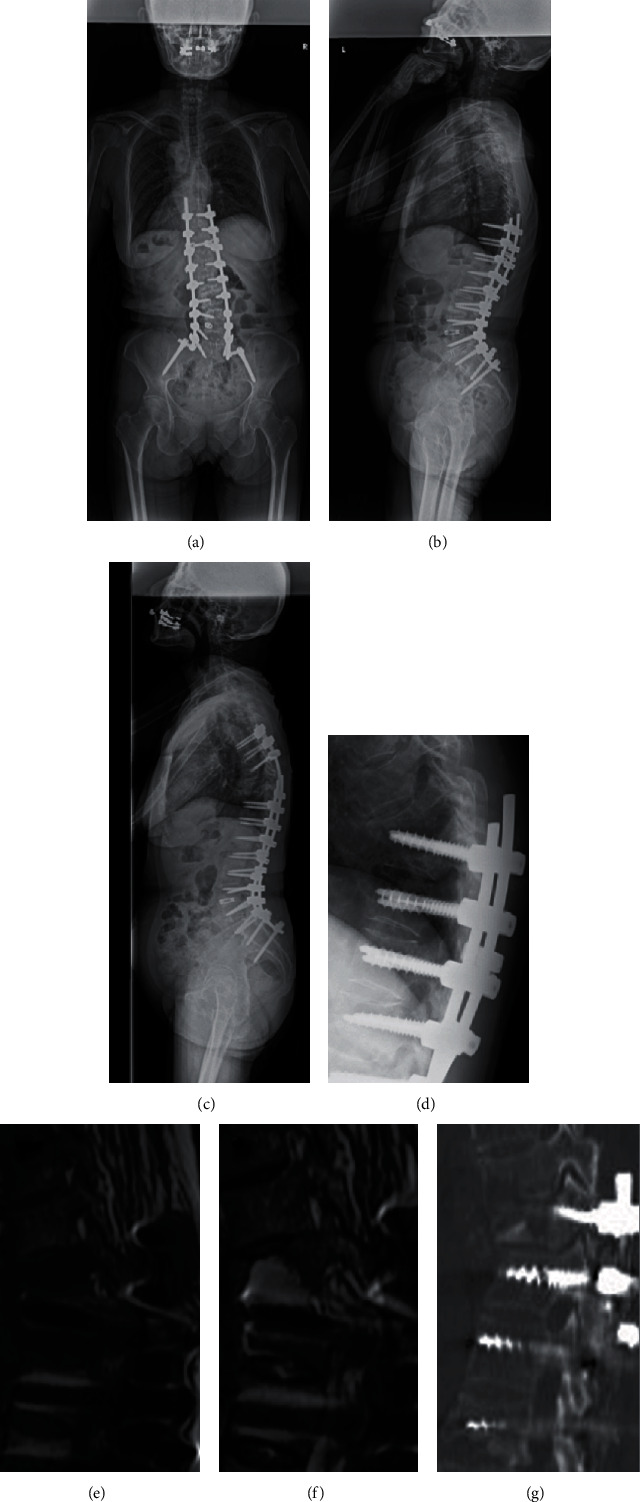
The 72-year-old female patient received PSF (Th10 to ilium). (a) Immediate postoperation whole spine posterior-anterior (PA) standing radiograph demonstrates normal coronal alignment. (b) Immediate postoperation whole spine lateral standing radiograph demonstrates normal sagittal alignment. (c) Immediate postrevision lateral view shows normal sagittal alignment. (d) PA view at readmission shows severe PJK (PJA 33.4 deg.). (e) T1-weighed MR image (T1WI) around the UIV area shows bone destruction of both UIV and UIV+1 vertebra. (f) T2WI around the UIV area shows fluid correction around the UIV vertebra. (g) CT scan around the UIV area shows bone destruction of both UIV and UIV+1 vertebra.

A 72-year-old female underwent posterior spinal fusion (PSF, Th10-ilium) for Schwab-SRS type L ASD (Figures 1(a) and 1(b)) [4]. The blood test showed normalization of the white blood cell count (WBC) and C-reactive protein (CRP) at 2 weeks after surgery. She developed proximal junctional kyphosis (PJK) due to Th9 vertebral fracture 2 months after surgery. PJK was maintained after the administration of teriparatide, and her pain improved 5 months after surgery. However, she had acute bilateral leg paralysis and was admitted to our hospital at 12 months after surgery (Figures 1(d)–1(g)). The WBC and CRP were also increased (WBC 11200/*μ*L, CRP 13.6 mg/dL). Notably, the right knee showed swelling upon admission, and purulent joint fluid was detected by aspiration. She was diagnosed with PJF accompanied by pyogenic spondylitis and knee arthritis. Combined anterior/posterior (A/P) spinal fusion (extended fixation to Th5) was performed, and continuous irrigation and drainage of the right knee joint were started. Aggressive, meticulous surgical debridement of all devitalized tissue was performed. The pus was completely removed, and all necrotic tissues were extensively resected, including the cyst wall around the abscess. Bacterial cultures and drug sensitivity tests of the necrotic tissue and bone from the Th9 vertebra through the cleft were performed to prescribe accurate postoperative antibiotic therapy. Due to the significant destruction of the Th9 vertebral body, additional anterior vertebrectomy of Th9 and fusion using iliac crest bone was performed 2 days after the initial emergency surgery. Since methicillin-resistant Staphylococcus aureus (MRSA) was detected by both spine and knee cultures, vancomycin and cefazolin were continuously administered for 76 days after admission, when both the WBC and CRP levels returned to normal. She gradually recovered motor function and was discharged 102 days after admission (Figure 1(c)).

### 2.2. Case 2

**Figure 2:**
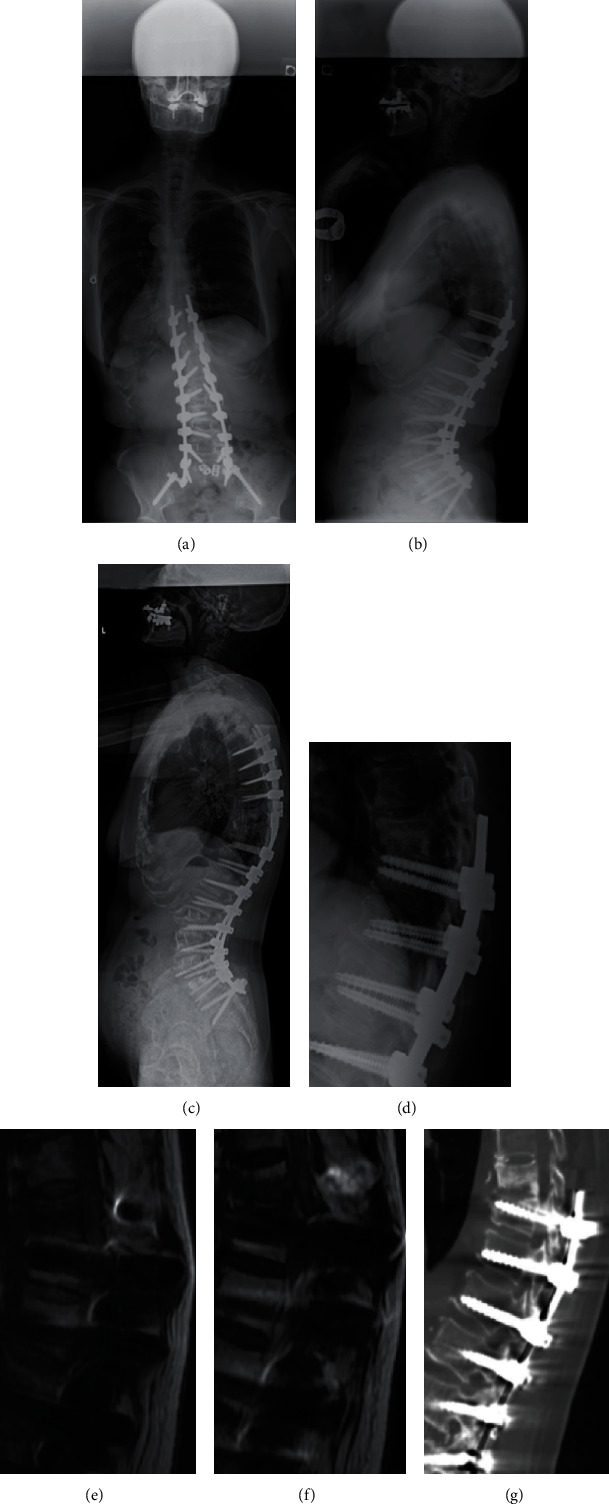
The 76-year-old female patient received PSF (Th10 to ilium). (a) Immediate postoperation whole spine PA standing radiograph demonstrates normal coronal alignment. (b) Immediate postoperation whole spine lateral standing radiograph demonstrates normal sagittal alignment. (c) Immediate postrevision whole spine lateral standing radiograph demonstrates normal sagittal alignment. (d) PA view at readmission shows moderate PJK (PJA 18.2 deg.). (e) T1WI around the upper instrumented vertebra (UIV) area shows bone destruction of the UIV vertebra. (f) T2WI around the UIV area shows fluid correction around the UIV+1 vertebra. (g) CT scan around the UIV area shows bone destruction of both UIV and UIV+1 vertebra.

A 76-year-old female underwent PSF (Th10-ilium) for Schwab-SRS type L ASD (Figures 2(a) and 2(b)), the postoperative course was fine at the beginning, and we had kept follow-up at the outpatient clinic. However, she was readmitted to our hospital due to high fever and bilateral motor weakness 13 months after surgery (Figures 2(d)–2(g)). The CT images revealed Th9 fracture, and MR images indicated severe spinal stenosis at the Th8/9 level. Combined A/P spinal fusion was performed. Aggressive, meticulous surgical debridement of all devitalized tissue was performed. The pus was completely removed, and all necrotic tissues were extensively resected, including the cyst wall around the abscess. We removed the Th10 pedicle screw and added fixation from the Th5-Th8 level so as to obtain firm stability to avoid further neurological damage. Since Staphylococcus epidermidis was detected from the intraoperative specimen of the Th9 vertebra, she was diagnosed with PJF accompanied by pyogenic spondylitis. Based on bacterial culture and drug sensitivity test, teicoplanin was continuously administered for 55 days after admission when the CRP level became normal. She gradually recovered motor function (Figure 2(c)).

## 3. Discussion

In this report, both patients developed pyogenic spondylitis more than 1 year after surgery. Delayed onset adjacent level pyogenic spondylitis is an extremely rare phenomenon, and only one study has been published. Nagoshi et al. reported that 2 of 3 cases required surgical treatment rather than antibiotic administration [5]. Adjacent segment infection after instrumented spinal surgery is generally a rare manifestation, and 3 studies have been reported [6–8]. All of the patients in the studies required surgical intervention in addition to the intravenous antibiotic treatment [6–8]. In the present study, considering the timing of the onset of spondylitis, it can be estimated that our cases were not surgical site infections but rather secondary infections that occurred after PJK. The pyogenic spondylitis developed adjacent to the UIV in both cases but not to the remote segment; thus, it can be considered as the delayed surgical site infection. However, we did not dissect the UIV+1 vertebra as well as the adjacent intervertebral disc space during surgery to minimize the risk of developing PJK. Additionally, if the infections were SSI, the infection did not retain into the UIV+1 vertebra but spread throughout the wound. In both cases, the wound was completely intact, and the infections were retained in the UIV+1 vertebra. Taken together, we believed that it is more suitable to recognize the infections not SSI by second infection. We do not have a clear explanation for this pathology. Bose et al. have described three possible causes of a delayed infection after instrumented spine surgery including intraoperative seeding, metal fretting, or micromotion between the implants causing a sterile inflammatory response or stimulating low-virulent organisms to fester and hematogenous seeding [9]. Hematogenous seeding may cause delayed infection especially in patients with distant infection foci such as our first case. In the presence of bacteremia, bacteria-related adherence and growth around the instrument are common sequelae. Since the delayed infections of our 2 cases developed not in the fused segments but in the adjacent vertebra, the cause of the first case can be speculated as stimulation of low-virulent organisms to fester and hematogenous seeding and that of the second case as metal fretting and a sterile inflammatory response causing hematogenous microbial seeding, respectively. Additional studies on this phenomenon are warranted to elucidate the pathogenesis of this complication.

## 4. Conclusion

Delayed onset adjacent level pyogenic spondylitis is an extremely rare phenomenon. In this report, we described 2 rare cases of PJF accompanied by delayed infection following posterior spinal fusion for adult spinal deformity. This report suggested that late-onset infection can occur after the development of PJF in ASD surgery.

## Data Availability

The data used to support the findings of this study are included within the article.
